# Overexpressed L20 Rescues 50S Ribosomal Subunit Assembly Defects of *bipA*-Deletion in *Escherichia coli*

**DOI:** 10.3389/fmicb.2019.02982

**Published:** 2020-01-09

**Authors:** Eunsil Choi, Hyerin Jeon, Jeong-Il Oh, Jihwan Hwang

**Affiliations:** Department of Microbiology, Pusan National University, Busan, South Korea

**Keywords:** GTPase, ribosome assembly, BipA, 50S ribosomal subunit, L20 ribosomal protein, cold shock

## Abstract

The BipA (BPI-inducible protein A) protein is highly conserved in a large variety of bacteria and belongs to the translational GTPases, based on sequential and structural similarities. Despite its conservation in bacteria, *bipA* is not essential for cell growth under normal growth conditions. However, at 20°C, deletion of *bipA* causes not only severe growth defects but also several phenotypic changes such as capsule production, motility, and ribosome assembly, indicating that it has global regulatory properties. Our recent studies revealed that BipA is a novel ribosome-associating GTPase, whose expression is cold-shock-inducible and involved in the incorporation of the ribosomal protein (r-protein) L6. However, the precise mechanism of BipA in 50S ribosomal subunit assembly is not completely understood. In this study, to demonstrate the role of BipA in the 50S ribosomal subunit and possibly to find an interplaying partner(s), a genomic library was constructed and suppressor screening was conducted. Through screening, we found a suppressor gene, *rplT*, encoding r-protein L20, which is assembled at the early stage of ribosome assembly and negatively regulates its own expression at the translational level. We demonstrated that the exogenous expression of *rplT* restored the growth of *bipA*-deleted strain at low temperature by partially recovering the defects in ribosomal RNA processing and ribosome assembly. Our findings suggest that the function of BipA is pivotal for 50S ribosomal subunit biogenesis at a low temperature and imply that BipA and L20 may exert coordinated actions for proper ribosome assembly under cold-shock conditions.

## Introduction

Ribosome biogenesis is a highly complex process with chains of events including transcription, processing, and modifications of ribosomal RNAs (rRNAs) and ribosomal proteins (r-proteins), and the assembly of dozens of r-proteins with rRNAs (Wimberly et al., 2000). A number of ribosome assembly factors are also involved in and accelerate this process by facilitating correct rRNA folding and interactions between rRNAs and r-proteins and by serving as sensors of checkpoints during the assembly process or under various growth conditions. The ribosome assembly factors identified in *Escherichia coli* include GTPases (Der, Era, and ObgE), RNA helicases (DeaD, SrmB, and DbpA), protein chaperones (DnaK, GroEL, RimM, and RbfA), and RNA-modifying enzymes (KsgA and RrmJ) (Bylund et al., 1998; Caldas et al., 2000; El Hage et al., 2001; Maki et al., 2002; Charollais et al., 2003, 2004; Sharma et al., 2005; Hwang and Inouye, 2006; Jiang et al., 2006; Connolly et al., 2008; Elles et al., 2009). Nevertheless, there is no bacterial assembly factor whose role in the assembly process has been definitively elucidated, except for rRNA- or r-protein-modifying enzymes (Caldas et al., 2000; Gutgsell et al., 2005).

BipA is a highly conserved GTP-binding protein in almost all bacteria and has also been identified in the chloroplast of the halophytic plant *Suaeda salsa* (Wang et al., 2008). Despite its extensive conservation, it is not essential for survival under normal growth conditions (Margus et al., 2007; Krishnan and Flower, 2008). However, at low temperature, BipA is required for normal growth (Pfennig and Flower, 2001; Choi and Hwang, 2018) and, accordingly, the transcription of *bipA* is upregulated by cAMP–CRP complex at a low temperature (Choi and Hwang, 2018). In addition to growth at a low temperature, BipA is also involved in virulence and stress adaptation. Deletion of *bipA* affects pathogenicity, regulation of capsule synthesis, and flagella-mediated motility, suggesting that BipA may function as a regulator of a variety of virulence factors (Farris et al., 1998; Rowe et al., 2000; Grant et al., 2003; Duo et al., 2008). Furthermore, in both pathogenic and non-pathogenic bacteria, BipA is implicated in resistance to other stress factors such as antimicrobial peptides, antibiotics, acidic conditions, and detergent stress (Qi et al., 1995; Farris et al., 1998; Kiss et al., 2004; Duo et al., 2008; Neidig et al., 2013). Even in chloroplasts of *S. salsa*, the expression of *bipA* is upregulated by a variety of external stressors, and BipA plays an important role in obtaining tolerance to oxidative stresses (Wang et al., 2008).

Another functional aspect of BipA is that it can associate with the ribosome and shares structural similarity with translational GTPases (trGTPases) including IF2, EF-G, EF-Tu, and LepA (Ero et al., 2016). EF-G is arranged with domains G, G′, II, III, IV, and V (AEevarsson et al., 1994; Czworkowski et al., 1994), and all of the above-mentioned trGTPases contain domains G and II. Among them, EF-G, BipA, and LepA share domains III and V, although LepA and BipA lack the part equivalent to domain IV of EF-G but have a unique C-terminal domain (CTD). In addition, these trGTPases exhibit GTP-dependent ribosome association and their functional properties depend on GTPase activities stimulated by ribosome association (Qin et al., 2006; deLivron et al., 2009). Similarly, BipA also interacts with ribosomes only in the GTP-bound state, and is released from ribosomes while hydrolyzing GTP to GDP (deLivron and Robinson, 2008). Like other trGTPases, the GTPase activity of BipA is enhanced upon association with ribosomes (deLivron et al., 2009). Despite its similarity with trGTPases, the most evident role of BipA is as a 50S ribosomal subunit assembly factor. As we showed in a previous report, an in-frame deletion of *bipA* caused severe defects in ribosome assembly at a low temperature, leading to the accumulation of pre-50S particles lacking r-protein L6 (Choi and Hwang, 2018). Interestingly, it was revealed that BipA is a bifunctional GTP-binding protein, having molecular chaperone activity through its G-domain. This chaperone activity was independent of nucleotide and GTP hydrolysis activity (Choi and Hwang, 2018). Thus, we previously proposed that after association of BipA in a GTP-bound form with ribosome, its activity as a chaperone may promote the correct incorporation of r-protein L6 to the 50S ribosomal subunit under given conditions (Choi and Hwang, 2018). Another line of evidence for this is that deletion of *rluC* suppresses the growth defects of a *bipA*-deleted mutant at low temperature (Krishnan and Flower, 2008), whose gene product is a pseudouridine synthase modifying nucleotides U955, Y2504, and U2580 in 23S rRNA (Conrad et al., 1998). Therefore, there is very convincing evidence that BipA plays a pivotal role in 50S ribosomal subunit assembly using its chaperone activity at low temperature. However, despite this accumulated evidence, the systematic mechanism by which BipA promotes 50S ribosomal subunit assembly has not been elucidated yet.

In this study, to gain further understanding of the role of BipA in ribosome biogenesis, we performed genomic library screening at a non-permissive temperature and obtained 26 library clones suppressing the phenotype of *bipA*-deleted strain. Among them, we identified the *rplT* gene as partially recovering cold-sensitive growth and ribosome defects observed in the *bipA*-deleted strain. Through mutational analysis, we verified that the ribosome assembly activity of L20 was responsible for the suppression.

## Materials and Methods

### Bacterial Strains and Growth Conditions

The *E. coli* strains used in this study are listed in Table 1. Those strains were grown in Luria–Bertani (LB) medium with chloramphenicol (50 μg/ml), kanamycin (50 μg/ml), or ampicillin (100 μg/ml) as needed.

**TABLE 1:** Strains used in this study and their genotypes.

**Strains**	**Description**	**Source or references**
MG1655	F^–^ lambda^–^ *ilvG*^–^ *rfb*-*50 rph*-*1*	Blattner et al., 1997
DH5α	F^–^ Φ80*lac*ZΔM15 Δ(*lac*ZYA-*arg*F) U169 *recA*1 *endA*1 *hsdR*17(rk^–^,mk^+^) *phoA supE*44 *thi*-1 *gyrA*96 *relA*1 λ^–^	Gibco-BRL
ESC19	*bipA*:kan, MG1655	Choi and Hwang, 2018
ESC29	*lacZ*:kan, MG1655	This study

For construction of the *lacZ*-deleted strain (ESC29), *lacZ*:kan DNA fragments were amplified by PCR using pKD13 as a template and primer sets (Tables 2, 3; Datsenko and Wanner, 2000). Then, strain MG1655 harboring pKD46 was subjected to linear DNA transformation (Datsenko and Wanner, 2000). Transformants resistant to kanamycin were isolated and confirmed to have undergone gene disruption by PCR.

**TABLE 2:** Primers used in this study and their sequences.

**Primer name**	**Sequence (5′→3′)**	**Source or references**
**Construction of deletion strain**		
lacZ-del-F	GGAATTGTGAGCGGATAACA ATTTCACACAGGAAACAGC TGTGTAGGCTGGAGCTGCTTC	This study
lacZ-del-R	TTACGCGAAATACGGGCA GACATGGCCTGCCCGGTTA TTAATTCCGGGGATCCGTCGACC	This study
lacZ-150-U	CACGACAGGTT TCCCGACTG	This study
lacZ-150-D	TCGGGAAAAACG GGAAGTAGG	This study
**Cloning of subclones of suppressors**		
BIS02-F-*Bam*HI	TAGGATCCGATCATG GTGCTCGCTC	This study
BIS02-1-R-*Bam*HI	GCGGATCCATG TGCTCTCCTGTATC	This study
BIS02-2-R-*Bam*HI	ACGGATCCTG CTTGCGTTACCTTTTG	This study
**Construction of *rplT* mutant clone**		
rplT-C1-L-3	GAACGTGGTC AACAATCGCC	This study
rplT-C1-R-5	GTGGTTGACGC ATTTGACGG	This study
BIS02-2-L-3	CAACGCGGTATACGCG TTTTACGCGAGCCATA	This study
BIS02-2-R-5	TATGGCTCGCGTAAAA CGCGTATACCGCGTTG	This study
rplT-R50AR51A-F	TTACCGTGACGC TGCTCAACGTAAGCGTCAG	This study
rplT-R50AR51A-R	CTTACGTTGAGC AGCGTCACGGTAAGCATAC	This study
rplT-R92AK93A-F	TGAAATCGACGC TGCGATCCTGGCTGATATC	This study
rplT-R92AK93A-R	AGCCAGGATCGC AGCGTCGATTTCAACAGAG	This study
**Construction of *lacZ*-promoter fusion clones for β-galactosidase assay**		
P*_*rpmI*_*-5-*Eco*RI	GTGAATTCGTAGAC TTAGTCGAGATC	This study
P*_*rpmI*_*-3-*Bam*HI	TAGGATCCACCAT GGCTTTCGGAC	This study
**qRT-PCR**		
rrlA-RT-F	ATATTCCTG TACTTGGTGTT	This study
rrlA-RT-R	CTTGGTATT CTCTACCTGAC	This study
P23S-U-5	ATCTTCGGGT TGTGAGG	This study
P23S-U-3	GGAATCTCGG TTGATTTC	This study
P23S-D-5	CTAGTACGAG AGGACCGG	This study
P23S-D-3	CGGCGTTGTA AGGTTAAG	This study
rrsA-RT-5	GACTTGGAG GTTGTGCCCTT	Choi and Hwang, 2018
rrsA-RT-3	GATAAGGGTT GCGCTCGTTG	Choi and Hwang, 2018
P16S-U-5	GTGGGCACTCG AAGATACGG	This study
P16S-U-3	TCTTGCGACGT TATGCGGT	This study
P16S-D-5	GAGAGCAAGC GGACCTCATA	This study
P16S-D-3	TGTGAGCAC TTCAAAGAACGC	This study

*The solid underlines are the sites of the listed restriction enzymes.*

**TABLE 3:** Plasmids used in this study and their genotypes.

**Plasmids**	**Description**	**Source or references**
pACYC184	Cm^R^, Tc^R^, *ori* p15A, cloning vector	New England Biolabs
pRS414	*bla*-Tl_4_-*Eco*RI-*Sma*I-*Bam*HI *lac*Z′	Simons et al., 1987
pKD46	*repA101*(ts) *oriR101 bla P_*araB*_*−(γβ *exo*)	Datsenko and Wanner, 2000
pKD13	*oriR*_γ *R*6*k*_ *bla* FRT:kan:FRT	Datsenko and Wanner, 2000
pACYC184BipA	*bipA*^+^, pACYC184	This study
pBIS01	*infC^+^-rpmI^+^-rplT^+^-pheM^+^*, pACYC184	This study
pBIS02	*rpmI^+^-rplT^+^-pheM^+^*, pACYC184	This study
pBIS02-1	*rpmI* ^+^, pACYC184	This study
pBIS02-2	*rpmI^+^-rplT ^+^*, pACYC184	This study
pBIS02-2ΔN	*rpmI^+^-rplT* (Δ6-29 a.a), pACYC184	This study
pBIS02-2ΔC	*rpmI^+^-rplT* (Δ60-118 a.a.), pACYC184	This study
pBIS02-2NM	*rpmI*^+^-*rplT*_R50AR51A_, pACYC184	This study
pBIS02-2CM	*rpmI*-*rplT*_R92AK93A_, pACYC184	This study
pRS414-P*_*rpmI*_*	*rpmI*^+^ regulatory region-*lac*Z’, pRS414	This study

### Construction of an *E. coli* Genomic Library

Genomic DNA (gDNA) from *bipA*-deleted cells (ESC19), which was constructed in our previous study, was used for the library construction (Choi and Hwang, 2018). gDNA was purified as previously described (Silhavy et al., 1984) and 0.1 mg of gDNA was partially digested with *Sau*3AI. Then, the digested gDNA fragments were resolved by 0.8% agarose gel electrophoresis and the fragments of 1–6 kb were extracted from the gel. Thereafter, the purified DNA fragments of varying sizes were ligated into pACYC184 linearized and dephosphorylated with *Bam*HI and calf intestine alkaline phosphatase (CIAP), respectively. The ligation reaction mixtures were transformed into DH5α cells and the transformants were spread on LB agar plates containing chloramphenicol. After overnight incubation, all colonies were scraped and collected. Genomic library clones were extracted from those collected cell pellets, yielding the ESC19 genomic library.

### Suppressor Screening in the Deletion of *bipA*

To screen suppressors of the deletion of *bipA*, ESC19 cells were transformed with the ESC19 genomic library and transformants were incubated on LB agar plates containing chloramphenicol and kanamycin at 20°C. Suppressor screening was based on colony size, and larger colonies than those harboring pACYC184 were selected as positive suppressors. Plasmids were extracted from those positive colonies, and the purified plasmids were retransformed into ESC19 cells to confirm their abilities to suppress the cold-sensitivity of strain ESC19. A total of 26 library clones could consistently suppress the growth defect of ESC19 at 20°C, and were sequenced from both ends to identify the inserted gDNA fragments.

To identify the exact gene responsible for the suppression of deletion of *bipA*, derivatives of positive suppressor were sub-cloned into pACYC184. Truncated inserts were amplified by PCR using pBIS02 as a template and the primer sets listed in Table 2. The PCR fragments were ligated into the *Sma*I site of pUC19, followed by digestion with *Bam*HI. Each insert was ligated into the *Bam*HI site of pACYC184, yielding pBIS02-1 and pBIS02-2. The original suppressor library clones and their derivatives are listed in Table 3.

### Plasmid Construction

To construct truncation mutants of *rplT*, DNA fragments of truncated *rplT* were amplified by PCR using pBIS02-2 as a template and the primer sets presented in Table 2. Each PCR fragment was subjected to blunt-end ligation into pUC19 linearized with *Sma*I and dephosphorylated with CIAP. Then, these subclones were digested with *Bam*HI and each insert was ligated into the same site of pACYC184, yielding pBIS02-2ΔN and pBIS02-2ΔC. Point mutations in *rplT* were introduced by site-directed mutagenesis PCR using pBIS02-2 as a template and the primer sets listed in Table 2, yielding pBIS02-2NM and pBIS02-2CM.

To generate a *lacZ*-fused regulatory region of the *rpmI* gene, the regulatory region of *rpmI* was amplified by PCR using MG1655 as a template and the primers P*_*rpmI*_*-5-*Eco*RI and P*_*rpmI*_*-3-*Bam*HI. Then, PCR fragments were ligated into the *Sma*I site of pUC19. This subclone was digested with the *Bam*HI and *Eco*RI restriction enzymes. Then, the insert was ligated into pRS414 vectors digested with *Bam*HI and *Eco*RI, yielding pRS414-P*_*rpmI*_*.

### β-Galactosidase Assay

ESC29 cells pre-transformed with pACYC184, pBIS02-2, or pBIS02-2 derivatives were transformed with pRS414-P*_*rpmI*_* and inoculated in 3 ml of LB medium containing ampicillin, chloramphenicol, and kanamycin. After incubation overnight at 37°C, cultures were diluted 100 times in LB medium containing antibiotics, after which they were further incubated at 20°C until the OD_600_ value reached ∼0.5–0.6. Cells were harvested by centrifugation, after which 4 μl of chloroform, 40 μl of 1% SDS, and 400 μl of Buffer Z [60 mM Na_2_HPO_4_⋅2H_2_O, 40 mM NaH_2_PO_4_⋅H_2_O, 10 mM KCl, 1 mM MgSO_4_⋅7H_2_O, and 50 mM β-mercaptoethanol (BME)] were added to each sample. The tubes containing the reaction mixtures were vortexed and incubated for 5 min at 37°C. Then, 80 μl of *ortho*-nitrophenyl-β-D-galactoside (ONPG, 4 mg/ml in H_2_O) was added. The mixture was then further incubated at room temperature for 10 min, at which time 160 μl of 1 M Na_2_CO_3_ was added to stop the reaction and the optical density was measured at 420 and 550 nm by using a Multiskan^TM^ GO Microplate Spectrophotometer (Thermo Fisher Scientific^TM^). The standardized amount of β-galactosidase activity was reported in Miller units (Miller, 1972). Each value was averaged from at least three independent experiments.

### Sucrose Density Gradient Sedimentation

Polysomes were prepared and resolved as described previously, with minor modification (Choi and Hwang, 2018). In brief, ESC19 transformants were grown at 20 or 37°C until an OD_600_ of ∼0.5–0.6 was obtained in 100 ml of LB medium supplemented with appropriate antibiotics. Polysomes were trapped by the addition of chloramphenicol to the culture at a final concentration of 100 μg/ml. Following an additional 3 min of incubation, the cells were harvested by centrifugation. The cell pellet was washed with 1 ml of Buffer BP (20 mM Tris–HCl (pH 7.5), 10 mM MgCl_2_, 100 mM NH_4_Cl, and 5 mM BME) and then resuspended in 0.5 ml of Buffer BP, followed by placement in a Beckman ultracentrifuge tube. Cells were then lysed by immersing the tube into a liquid nitrogen bath for 1 min, followed by thawing in cold water until no traces of ice remained. This freeze–thaw cycle was repeated twice, and the lysate was subsequently subjected to centrifugation at 100,000 × *g* for 10 min in a Beckman TLA100.4 rotor.

Then, 0.5 ml of cleared lysates was added to 10 ml of 5–40% sucrose gradient in Buffer BP and resolved by ultracentrifugation at 4°C in a Beckman SW41 rotor for 2.5 h at 37,000 rpm. For the analysis of ribosomal subunits, cell pellets were resuspended in 0.5 ml of Buffer BS [20 mM Tris–HCl (pH 7.5), 1 mM MgCl_2_, 100 mM NH_4_Cl, and 5 mM BME] and cleared cell lysates were subjected to 10 ml of 5–25% sucrose gradient in Buffer BS, followed by centrifugation at 4°C in a Beckman SW41 rotor for 3.5 h at 37,000 rpm.

### rRNA Analyses and Northern Blotting

Total RNAs were extracted from cells incubated at 37 or 20°C to an OD_600_ of 0.5–0.6 using the hot phenol method, as described previously (Sarmientos et al., 1983), and quantified by UV absorbance at 260 nm (NanoDrop^TM^ One^C^ Spectrophotometer, Thermo Fisher Scientific^TM^). For 23S rRNA analyses, 4 μg of RNA was separated on 1.2% agarose gel in 0.5× TBE [45 mM Tris-borate (pH 8.3), 1 mM ethylenediaminetetraacetic acid (EDTA)] and transferred overnight to nylon membranes (Hybond N+, Amersham) by capillary action. After transfer, both sides of the membrane were cross-linked by exposure to UV light using CL-1000 UV crosslinker (UVP Inc.). The membrane was prehybridized for 4 h at 65°C in hybridization buffer [100 mM sodium phosphate (pH 7.2), 0.2 mM EDTA, 1% SDS, and 1 mg/ml bovine serum albumin] with 50 μg/ml salmon sperm DNA, which was boiled for 10 min, followed by hybridizing in the same buffer containing 500 ng/ml of biotin-labeled probes described in Supplementary Table S1. Hybridization and washing conditions are presented in Supplementary Table S1. Biotin signals were detected using a Biotin Chromogenic Detection Kit (Thermo Fisher Scientific^TM^), following the manufacturer’s instructions. To visualize 16S and 17S rRNAs, 1 μg of total RNA was separated on 2% agarose gel electrophoresis in 1× TAE (40 mM Tris, 20 mM acetic acid, and 1 mM EDTA).

### Quantitative Real-Time PCR

MG1655, ESC19, and ESC19 transformants were grown in LB medium containing appropriate antibiotics at 37 or 20°C to OD_600_ = 0.5. Cells were equally harvested based on the OD value and then total RNAs were extracted as described previously (Sarmientos et al., 1983; Choi and Hwang, 2018). The quality and concentration of RNA were assessed using agarose gel electrophoresis and a Thermo Scientific^TM^ NanoDrop^TM^ One microvolume spectrophotometer, respectively. To remove contaminating gDNA, extracted RNAs were treated with RNase-free DNase (Qiagen), in accordance with the manufacturer’s instructions. After DNase treatment, the DNase was removed by phenol extraction. The quality and concentration of the purified RNA were assessed as described above. Then, complementary DNAs (cDNAs) were synthesized using 1 μg of total RNA and the BioFact^TM^ RT-Kit comprising Moloney murine leukemia virus RNaseH^–^ RTase. Quantitative Real-Time PCR (qRT-PCR) reaction was performed using 0.2 μl of cDNA and 2 pmole of each primer (Table 2) in a 20 μl volume with 2× SYBR Green Master Mix (Qiagen). The reactions were carried out on a QuantStudio 3 Real-Time PCR Instrument (Applied Biosystems) using the following cycling parameters: 95°C for 10 min, 40 cycles of denaturation at 95°C for 15 s, primer annealing at 50°C for 15 s, and extension at 72°C for 30 s. The 23S and 16S rRNAs served as references for normalization in calculating the relative expression levels of unprocessed 23S and 16S rRNA, respectively.

### Statistical Analysis

Results are presented as the mean ± SD of three independent experiments. The data were analyzed by unpaired two-way *t*-test. The statistical analyses were performed with the following significance levels: NS, non-significant; ^∗^*p* < 0.05; ^∗∗^*p* < 0.01; ^∗∗∗^*p* < 0.001.

## Results

### Multicopy Suppressors Rescue Growth Defect of a *bipA*-Deleted Strain at Low Temperature

**FIGURE 1:**
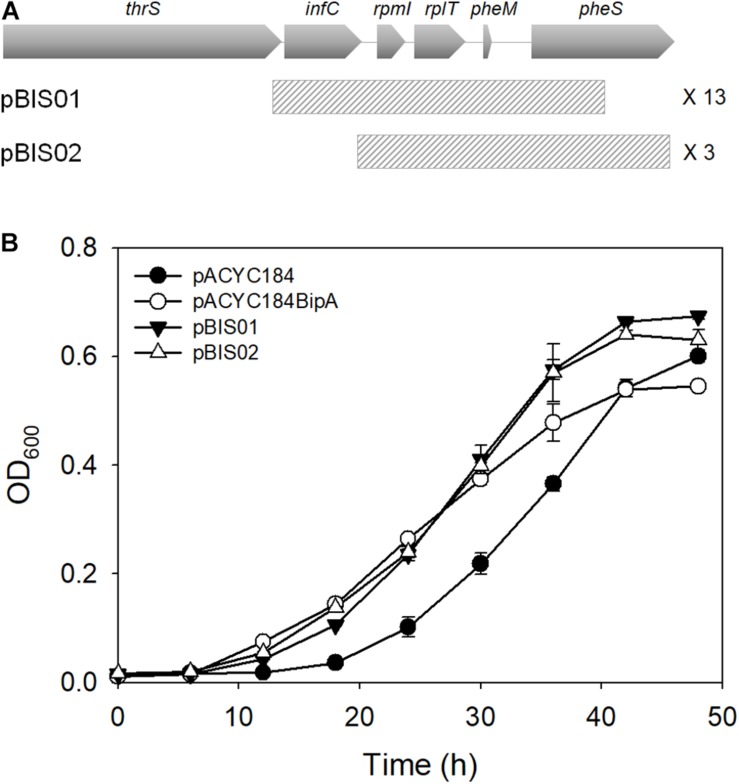
Isolation of library clones suppressing growth defects of the ESC19 strain at 20°C. **(A)** Schematic diagrams of genomic loci containing suppressor genes. The striped square boxes represent suppressor library clones. **(B)** Recovered growth phenotype of the ESC19 strain by isolated suppressors. ESC19 cells were transformed with each plasmid as presented above. Each transformant was incubated in LB medium containing chloramphenicol and kanamycin at 20°C. Cultures were diluted five times before measurement of the optical density at 600°nm. Experiments were performed in three independent repetitions and error bars represent SD.

In an attempt to further comprehend the function of BipA in *E. coli*, we constructed a genomic library and searched for an element(s) in the *E. coli* genome that can recover the growth defect of strain ESC19 (*bipA* deletion) at low temperature. First, a genomic library was constructed using gDNA from the strain ESC19, as described in the section “Materials and Methods.” The constructed genomic library was transformed into ESC19 cells and the transformants were tested for their abilities to grow normally at a non-permissive temperature (20°C) on LB plates containing chloramphenicol and kanamycin. Plasmid DNAs from those colonies gaining the ability to grow at 20°C were purified and reintroduced into ESC19 to confirm whether restoration was solely due to the presence of the selected plasmid. Approximately 10,000 transformants were screened and a total of 26 clones were isolated as possible positive candidates for the suppressor clones. The screened library clones were sequenced from both ends of the insert and sequence analysis revealed that, out of 26 clones, 16 contained the genomic fragment from *rpmI*, *rplT*, to *pheM* (pBIS01 and pBIS02) (Figure 1A). The rest 10 clones contained genomic fragments of the *hfq* open reading frame or the *yebC* locus. In this study, we focused on the most screened suppressors, pBIS01 and pBIS02; the rest are under further investigation (data not shown).

These suppressor clones were further confirmed by culturing ESC19 cells in liquid medium at low temperature. As shown in Figure 1B, both suppressor clones could recover the growth of ESC19 cells to the extent of isogenic wild-type ESC19/pACYC184BipA cells. Notably, the growth rate of suppressed cells appeared to be faster than that of the ESC19/pACYC184BipA cells after the mid-exponential phase.

**FIGURE 2:**
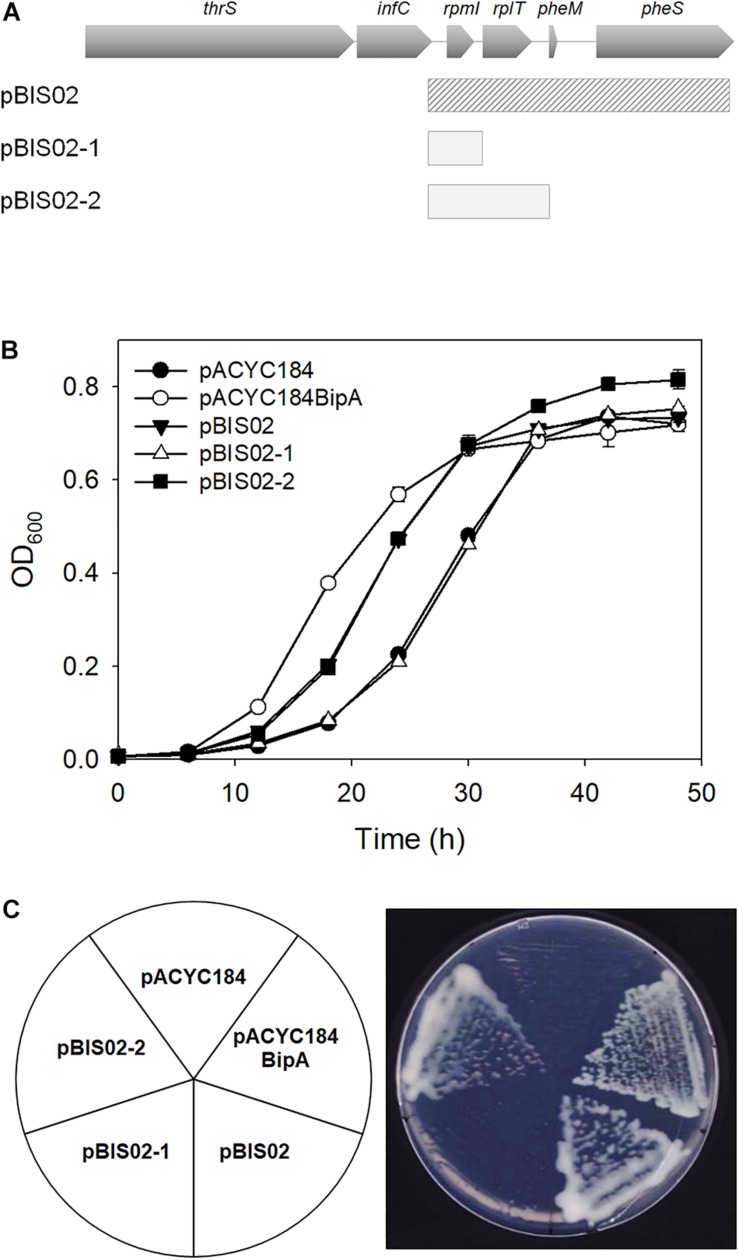
Identification of gene responsible for suppression. **(A)** Schematic diagrams of suppressor and sub-cloned plasmids. The striped square box represents pBIS02. The light gray boxes indicate sub-cloned plasmids derived from suppressor pBIS02. **(B,C)** Suppressive effects of pBIS02 and its derivatives. Growth of transformants was measured as described in Figure 1B. Experiments were performed in three independent repetitions and error bars represent SD. Overnight cultures of ESC19 cells harboring each plasmid presented above were diluted 10^3^-fold in LB medium, and a 2-μl aliquot of the diluted culture was streaked onto LB agar plates containing chloramphenicol and kanamycin, after which the plates were incubated at 20°C. Experiments were performed in three independent repetitions and error bars represent SD.

To identify the gene(s) responsible for suppression of the cold-sensitivity of the strain ESC19, two derivatives of pBIS02 were constructed by PCR using pBIS02 as a template: pBIS02-1 and pBIS02-2 (Figure 2A). ESC19 cells were transformed with pACYC184, pACYC184BipA, pBIS02, pBIS02-1, or pBIS02-2 and the growth of transformants was monitored at 20°C in LB medium containing chloramphenicol and kanamycin. L20 directly binds to the regulatory pseudoknot which is located close to the translation start site of *rpmI*, and this binding represses the translation of *rplT* as well as *rpmI* via translational coupling (Lesage et al., 1990; Guillier et al., 2005). Note that pBIS02 and its derivatives were lacking sequences required for forming pseudoknot, suggesting constitutive expression of *rplT* from pBIS02 and pBIS02-2. ESC19 cells harboring pBIS02-1 still showed cold-sensitivity, whereas, when transformed into ESC19, pBIS02-2 retained the ability to suppress the growth defect at 20°C, suggesting that the *rplT* gene encoding r-protein L20 is responsible for the suppression (Figures 2B,C). At the mid-exponential phase, the doubling time of the ESC19 expressing *bipA* was 3.72 h, which is marginally faster than that of ESC19 harboring pACYC184 (4.00 h) or pBIS02-2 (3.98 h). Notably, prolonged acclimation phase of the ESC19 cells with pACYC184 was shortened by expression of *bipA* or *rplT* (Figures 1B, 2B). This indicates that expression of BipA or L20 in the ESC19 cells promotes the entrance into exponential phase.

### The N-Terminal Domain of L20 Is Responsible for Suppression

**FIGURE 3:**
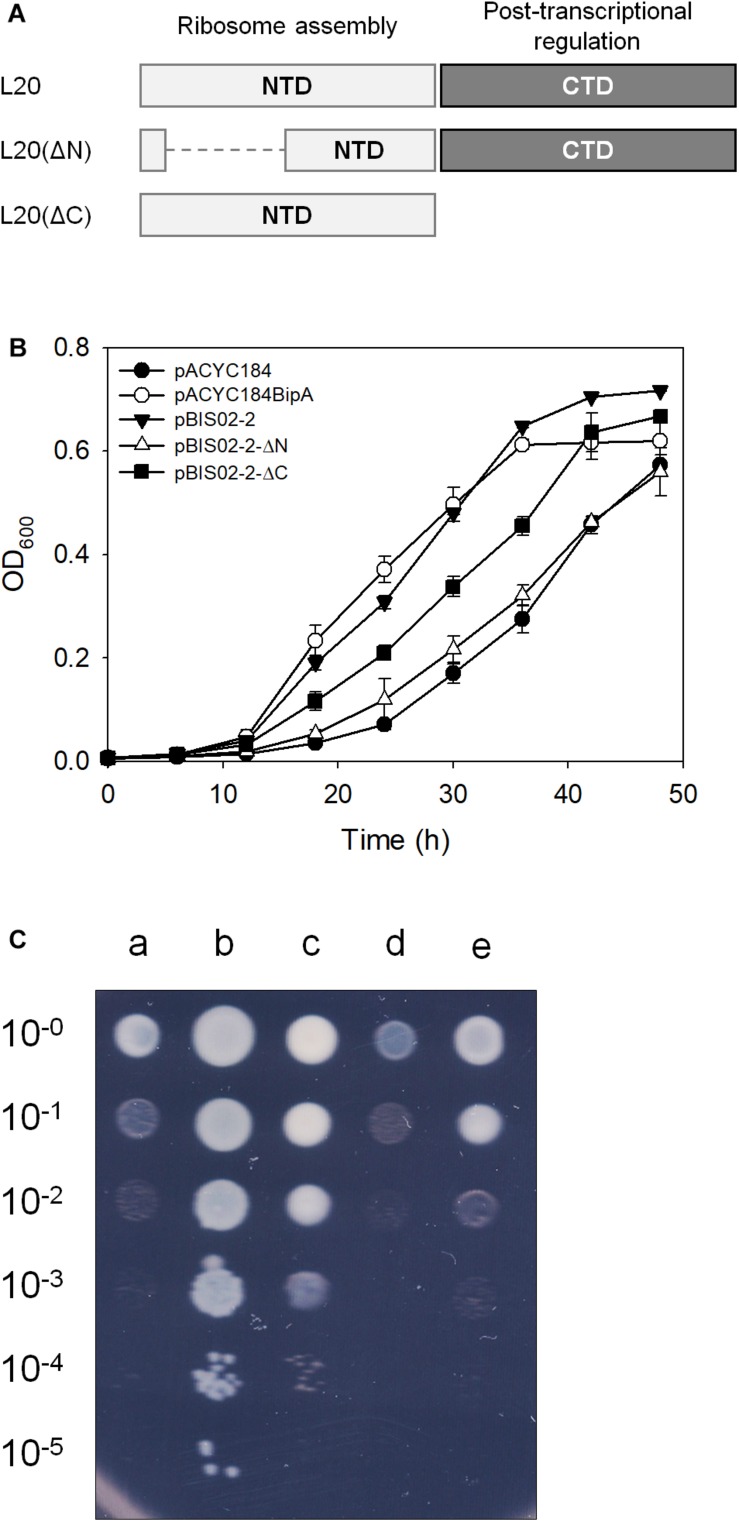
Suppressive activities of truncated mutant forms of L20. **(A)** Schematic illustration of L20 and its truncated mutants. The NTD and CTD of L20 are represented by light and dark gray boxes, respectively. Suppressive effects of the wild-type and truncated L20 on cold-sensitive phenotype of the ESC19 cells on liquid **(B)** and solid media **(C)**. Growth of transformants was measured as described in Figure 1B. Experiments were performed in three independent repetitions and error bars represent SD. For spotting assay, the overnight cultures of transformants were diluted to get OD_600_ of 0.2 and serially diluted 10-fold over the range of 10^–1^ to 10^–5^. Then, 3 μl aliquots were spotted on LB medium containing chloramphenicol and kanamycin, after which the plates were incubated at 20°C. a, pACYC184; b, pACYC184BipA; c, pBIS02-2; d, pBIS02-2ΔN; and e, pBIS02-2ΔC.

The L20 protein is one of the initiating components of 50S ribosomal subunit synthesis and consists of the linear N-terminal domain (NTD) and globular CTD, whose functions are also distinctively defined. The highly cationic NTD of L20 penetrates deeply into the 50S ribosomal subunit and interacts with helices 25, 40, and 41 of 23S rRNA. The CTD of L20 interacts with helices 40 and 41 of the 23S rRNA and with r-proteins L21 and L31 at the surface of the ribosome (Noller et al., 1981; Harms et al., 2001; Timsit et al., 2009). Truncation mutation in NTD leads to the accumulation of precursors of 50S ribosomal subunit (Guillier et al., 2005), suggesting its direct role as a ribosome assembly component. The CTD of L20 is responsible for binding to pseudoknot and negatively regulates expression of *rpmT* and *rplT* (Lesage et al., 1990). To further determine whether L20 suppresses the phenotype of ESC19 by being directly involved in the ribosome assembly or indirectly by regulating the expression of *rpmI* and *rplT*, truncation mutants of L20 were constructed as shown in Figure 3A. The recombinant protein, L20(ΔN) or L20(ΔC) having partial deletion at the N-terminus or truncation at the C-terminus was expressed in the ESC19 strain and growth curves were monitored at 20°C for 48 h. L20(ΔC), although not as much as the wild-type L20, restored the growth of the ESC19 strain, while the ESC19 strain expressing L20(ΔN) was more cold-sensitive than the strain expressing L20(ΔC) (Figure 3B), indicating that the suppressive effect may not be mediated by the downregulation of *rpmI* and *rplT*. These two mutant forms of L20 were tested for their translational repression functionality in the CTD. First, the upstream regulatory region of *rpmI* containing the L20-binding pseudoknot was amplified by PCR and ligated into the *lacZ* translational fusion vector pRS414, yielding pRS414-P*_*rpmI*_* (Supplementary Figure S1A). The ESC29 (*bipA* and *lacZ* deletion) strain pre-transformed with pRS414-P*_*rpmI*_* was transformed by pACYC184, pBIS02-2, pBIS02-2ΔN, or pBIS02-2ΔC. Then, the resulting transformants were incubated at 20°C to the exponential phase and harvested for β-galactosidase assay. As shown in Supplementary Figure S1B, compared with the activity in the pACYC184 transformant, plasmid-borne expression of the wild-type L20 significantly reduced the units of β-galactosidase from ∼1234 to 293. However, when only NTD was expressed, repression activity was not observed, with units of ∼1178, which was similar to that of the ESC29 strain with pACYC184. In contrast, the units of β-galactosidase obtained from the ESC29 strain with pBIS02-2ΔN amounted to ∼280, which was similar to that from the ESC29 strain with pBIS02-2. These results suggest that the regulatory domain in L20(ΔN) is soundly functional and the ribosome assembly activity still resides in the NTD of L20(ΔC), as shown in Figure 3B.

**FIGURE 4:**
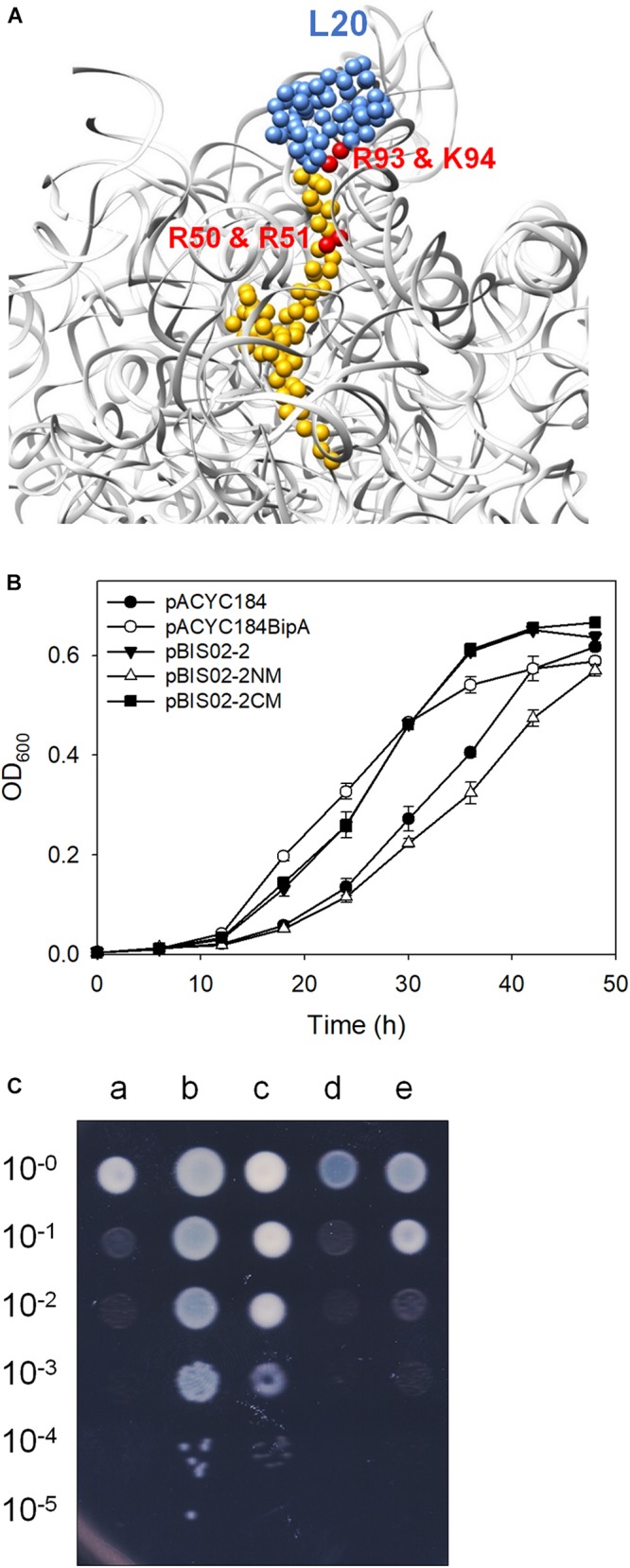
Mutational analysis of L20. **(A)** The interaction of L20 (blue and yellow) with 23S rRNA (light gray) (PDB entry 4V4A). The arginine and lysine residues interacting with 23S rRNA are highlighted in red. Growth restoration of ESC19 by overexpressed L20 and its mutants on liquid **(B)** and solid media **(C)**. Growth curves of transformants were measured as described in Figure 1B. Experiments were performed in three independent repetitions and error bars represent SD. For spotting assay, the transformants incubated at 37°C overnight were diluted and spotted on LB medium containing chloramphenicol and kanamycin as described in Figure 3C, after which the plates were incubated at 20°C. a, pACYC184; b, pACYC184BipA; c, pBIS02-2; d, pBIS02-2NM; and e, pBIS02-2CM.

As mentioned earlier, L20 interacts with 23S rRNA through conserved positively charged amino acids located in NTD (Figure 4A; Timsit et al., 2009). To further ascertain the suppressive ability of the NTD of L20, two arginine residues (50th and 51st a.a.) in the NTD and arginine and lysine residues (92nd and 93rd a.a.) in the globular CTD were mutated to alanine by site-directed mutagenesis using the primers shown in Table 2 and pBIS02-2 as a template, yielding pBIS02-2NM (expressing L20_R__50__AR__51__A_) and pBIS02-2CM (expressing L20_R__92__AK__93__A_). Then, growth curves of the ESC19 strains harboring pACYC184, pACYC184BipA, pBIS02-2, or pBIS02-2 derivatives were measured in LB medium containing chloramphenicol and kanamycin for 48 h. The ESC19 strain expressing L20_R__50__AR__51__A_ lost the suppressive activity with a slightly lower growth rate than that of the control strain transformed with pACYC184, whereas L20_R__92__AK__93__A_ still exerted the same effect as the wild-type L20 (Figure 4B). The suppressive effects of L20 mutants were also confirmed in solid medium. As shown in Figure 4C, the pattern of colony formation ability from the ESC19 strains harboring pBIS02-2, pBIS02-2CM, or pBIS02-2NM showed very similar suppressive effects as in Figure 3C, indicating the more important role of the NTD of L20 in the suppression. The expression and activity of these point-mutated L20 proteins were also verified by β-galactosidase assay using pRS414-P*_*rpmI*_*. L20_R__50__AR__51__A_ still had activity of translational repression, which was similar to that of the wild-type L20, whereas L20_R__92__AK__93__A_ appeared to show a decrease in such activity (Supplementary Figure S1C), suggesting that the L20 derivatives were expressed in the functional form. Compared with truncation mutation of the CTD, point mutation in the CTD did not completely inactivate the repressive activity of L20. This might have been because substitution mutations in two amino acid residues were insufficient to remove the regulatory activity. Our findings demonstrate that the globular CTD is dispensable for the suppressive phenotype; rather, the rRNA interaction of L20 via the NTD is more critical for the suppression.

### Overexpression of L20 Rescues Ribosome Assembly Defects in the ESC19 Strain

**FIGURE 5:**
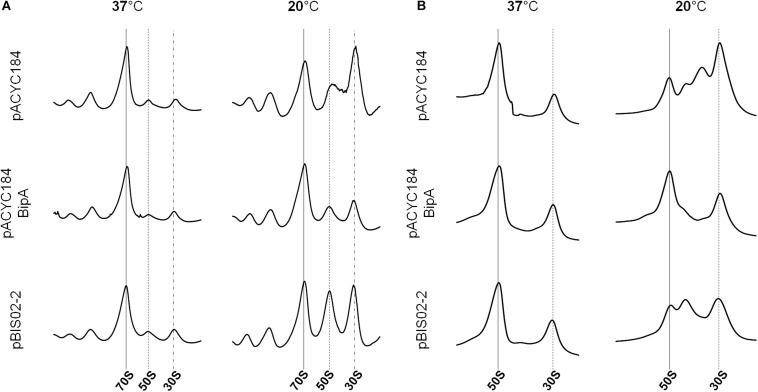
Restoration of ribosome assembly defects by the overexpression of suppressors. Polysome profiles **(A)** and subunit profiles **(B)** of ESC19 cells harboring pACYC184, pACYC184BipA, and pBIS02-2. Transformants were grown at 37 or 20°C in LB medium until reaching an OD_600_ of ∼0.6. Then, cell lysates for analysis were prepared and analyzed as described in the section “Materials and Methods.”

As we mentioned earlier, BipA plays various roles, such as in pathogenicity, capsule formation, and motility in cells. The most detrimental phenotype of deletion of *bipA* is cold-sensitivity caused by failure in 50S ribosomal subunit assembly. We have shown that the exogenous expression of *rplT* restored the normal growth of the ESC19 strain at low temperature. Thus, to demonstrate that the ribosomal defect of the ESC19 strain is suppressed by overexpressed L20, we analyzed polysomes and ribosomal subunits of the suppressed cells. The ESC19 cells harboring pACYC184, pACYC184BipA, or pBIS02-2 were incubated at 37 or 20°C, and the collected samples were subjected to sucrose gradient density sedimentation as described in the section “Materials and Methods.” At 37°C, the polysome and subunit profiles of all three transformants appeared to be normal with no aberrant particles (left panels in Figures 5A,B). On the other hand, at a non-permissive temperature, the ESC19 strain with pACYC184 cells accumulated significant levels of free 30S and 50S ribosomal subunits; it should be noted that the peak of 50S ribosomal subunit was almost half of that of 30S ribosomal subunit (right panel in Figure 5A). This was due to unprocessed precursors and/or destabilized 50S ribosomal subunits appearing between 50S and 30S peak positions in the subunit profile results (right panel in Figure 5B), as we reported previously (Choi and Hwang, 2018). The ESC19 strain with pBIS02-2 cells still showed abnormal 50S particles adjacent to the 50S position (right panel in Figure 5B), to a less severe extent than the ESC19 strain with pACYC184; owing to this partial restoration, high levels of free 50S and 30S ribosomal subunits remained in the polysome profile. Note that both normal and abnormal particles overlapped at the same position due to the higher sucrose gradient concentration and short sedimentation distance in the polysome profiling (right panel in Figure 5A). These results suggest that L20 can partially suppress the ribosomal defects of the ESC19 strain, alleviating the cold-sensitivity.

### Processing of rRNA Precursors in the Suppressor Strain

Next, since ribosomal defects are accompanied by incomplete rRNA processing, we examined whether the defect in rRNA processing is rescued by the overexpression of the screened suppressor. During 16S rRNA processing, 115 and 33 nt are removed from the 5′- and 3′-ends of the 16S rRNA precursors, respectively, which enables us to detect maturation status by agarose gel electrophoresis. On the other hand, mature 23S rRNA is produced by cleaving 7 and 8 nt at the 5′ and 3′-ends of pre-23S rRNA, respectively (Gutgsell and Jain, 2010). Therefore, to detect the unprocessed 23S rRNA, northern blotting was carried out. Their processing was also examined by qRT-PCR using primer sets amplifying the internal region of rRNA (16S-M and 23S-M) or 5′ or 3′-processing junctions (P16S-U or -D and 23S-U or -D, respectively) (Supplementary Figure S2). The extent of rRNA processing was calculated by dividing the value of the precursor by that of the internal region.

First, to verify whether precursors of rRNA are amassed in the ESC19 strain at low temperature, total RNAs extracted from the MG1655 and ESC19 cells incubated at 37 or 20°C were subjected to agarose gel electrophoretic analysis, northern blotting, and qRT-PCR. The results of the analyses demonstrate that deletion of *bipA* caused defects in the processing of both 16S and 23S rRNAs at low temperature (Supplementary Figure S3).

**FIGURE 6:**
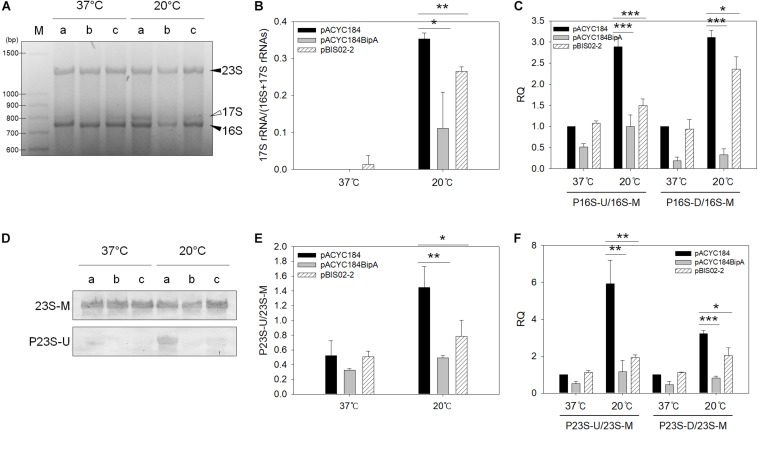
Analyses of 23S and 16S rRNAs processing. **(A)** Agarose gel electrophoresis analysis of 16S rRNA. One microgram of total RNA extracted from ESC19 cells transformed with (a) pACYC184, (b) pACYC184BipA, or (c) pBIS02-2 was subjected to 2.0% agarose gel electrophoresis, followed by ethidium bromide (EtBr) staining. Filled arrowheads indicate mature 16S and 23S rRNAs and open arrowhead indicates 16S rRNA precursor (17S). M, 100-bp DNA ladder (TaKaRa). Experiments were performed in three independent repetitions. **(B)** Densitometric analysis of 16S rRNA processing. The band intensity was quantified using the band analysis tools of Image Lab software version 5.2.1 (Bio-Rad). All data are expressed as the mean value of three independent experiments. qRT-PCR analyses of 16S **(C)** and 23S **(F)** rRNA maturation in ESC19 transformants. Total RNAs extracted from the same ESC19 transformants as in panel **(A)** were subjected to cDNA synthesis and qRT-PCR. The unprocessed 16S and 23S rRNA values were normalized to the values of total 16S and 23S rRNA, respectively. Experiments were performed in three independent repetitions. **(D)** Northern blot analysis of 23S rRNAs in ESC19 transformants. Four micrograms of total RNAs extracted from ESC19 cells harboring pACYC184, pACYC184BipA, or pBIS02-2 was separated on 1.2% agarose gel, followed by transfer to a nylon membrane. rRNAs were detected by biotin-labeled probes described in Supplementary Table S1. Northern blotting was performed in three independent repetitions. **(E)** Densitometric analysis of 23S rRNA processing. The blots were quantified using the gel analyzer function of ImageJ. Error bars represent SD. Statistical significance was derived from the unpaired two-tailed *t-*test. *NS*, non-significant; ^∗^*p* < 0.05; ^∗∗^*p* < 0.01; ^∗∗∗^*p* < 0.001.

Next, the ESC19 strains harboring pACYC184, pACYC184BipA, or pBIS02-2 were incubated at 37 or 20°C until an OD_600_ of 0.5–0.6 was reached. The total RNAs obtained from those strains were analyzed. As shown in Figure 6A, unprocessed 17S rRNA precursors were detected at 20°C from the ESC19 strain transformed with pACYC184, while reduced amounts of precursors were observed in the ESC19 strain with pACYC184BipA. The level of 17S rRNA in the ESC19 strain with pACYC184 cultured at 20°C was detected at a value of 0.35, while transformation of pACYC184BipA or pBIS02-2 led to a reduction of the level of 17S rRNA to 0.11 or 0.27, respectively, suggesting that exogenously expressed L20 partially promoted 17S rRNA processing at low temperature (Figure 6B). The recovery of 16S rRNA processing was also examined by qRT-PCR. Comparison of the P16S-U/16S-M and P16S-D/P16S-M ratios at 37 and 20°C in the ESC19 strain with pACYC184 showed a significant increase of the amount of 17S rRNA precursors with unprocessed ends at 20°C (P16S-U, ∼2.89-fold; P16S-D, ∼3.10-fold) (Figure 6C). BipA overexpressed from pACYC184BipA at 20°C recovered the defect in 16S rRNA maturation, reducing the P16S-U/16S-M and P16S-D/P16S-M ratios to ∼1.00-fold and ∼0.33-fold, respectively, which were very close to those of the same strain cultured at 37°C (P16S-U, ∼0.52-fold; P16S-D, ∼0.19-fold). By expressing L20, the P16S-U/16S-M ratio was decreased to ∼1.49-fold; however, the P16S-D/16S-M ratio remained quite high, at 2.36-fold. This was probably due to the high level of 30S ribosomal subunits existing as free ribosomal subunits (Figure 5A).

To examine the unprocessed 23S rRNA, northern blot analysis was carried out using RNAs, as shown in Figure 6A. As shown in Figure 6D, a tiny amount of unprocessed 23S rRNAs was observed in the ESC19 strain with pACYC184BipA cultured at 20°C (0.49), while the ESC19 strain with pACYC184 incubated at 20°C accumulated 23S rRNA precursors with unprocessed 5′-end (1.45). As expected, partial processing of 23S rRNAs was also observed in the ESC19 strain harboring pBIS02-2 (0.78), indicating a suppressive effect (Figure 6E). The same results were obtained from qRT-PCR. A significant increase in the level of 23S rRNA precursor was observed in the ESC19 strain with pACYC184 cultured at 20°C (P23S-U, 5.93-fold; P23S-D, 3.22-fold) (Figure 6F). This defect in 23S rRNA processing was recovered by pACYC184BipA, resulting in a decrease in the amount of 23S rRNA precursor (P23S-U, ∼1.16-fold; P23S-D, ∼0.81-fold). The ESC19 strain carrying pBIS02-2 also showed the reduced P23S-U/23S-M and P23S-D/23S-M ratios (∼1.93-fold and ∼2.04-fold, respectively). However, the levels of precursors were higher than those in pACYC184BipA transformant, indicating that L20 partially alleviated the defect in 23S rRNA processing. Taking these findings together, L20 overexpressed in the ESC19 strain promoted the maturation of both 23S and 16S rRNAs.

## Discussion

In this study, we performed genomic library screening, which revealed that overexpression of r-protein L20 ameliorated defects in growth and ribosome assembly of the ESC19 strain at low temperature. L20 consists of two structurally distinct domains, a linear NTD and a globular CTD (Harms et al., 2001). The CTD of L20 interacts with the helix 40–41 junction in domain II of 23S rRNA, whereas the NTD of L20 makes multiple contacts with domains I and II of 23S rRNA. L20 also plays a role as a translational repressor. In *E. coli*, the *rpmI* and *rplT* genes are arranged as an operon. L20 directly binds to *rpmI* mRNA and represses the expression of *rpmI* at the translational level (Lesage et al., 1990). As a result, the expression of *rplT* is also consequently repressed via translational coupling (Lesage et al., 1992). This negative translational regulation is mediated by the CTD of L20, indicating that these two binding sites of L20 in the leader sequence of *rpmI* mRNA and 23S rRNA are structurally similar (Guillier et al., 2002). Thus, our results from the truncated and point-mutated L20 revealed that the NTD, not the CTD, is essential for suppressing cold-sensitivity of the ESC19 strain. This implies that the exogenously expressed L20 functions as a ribosomal component rather than a translational regulator in the suppression of the *bipA* deletion phenotype, and that the suppression may not result from negatively affected ribosomal proteins and/or 50S ribosome biogenesis.

*In vitro*, L20 is absolutely required for the total reconstitution of active 50S ribosomal particles using rRNAs and r-proteins, whereas incubation of 50S ribosomal subunits with 4.3 M LiCl results in a 4.3c core (approximately 41S), which lacks L20 (Homann and Nierhaus, 1971). The partial reconstitution starting from the 4.3c core does not need L20. The later addition of L20 to this 4.3c core was shown to improve the stability of the 4.3c core. Furthermore, the 50S particles from the partial reconstitution are functionally active in terms of peptidyltransferase (Nowotny and Nierhaus, 1980). It should be noted that, although L20 is an early assembly r-protein, the stability of the 4.3c core lacking L20 can be obtained by the later addition of L20. Another aspect of L20 is that it can replace the function of L24, which initiates 50S ribosomal subunit assembly at a very early step (Spillmann et al., 1977; Franceschi and Nierhaus, 1988). The presence of L24 accelerates the overall rate of ribosome assembly by reducing the activation energy of the rate-limiting step of the initial assembly step (Herold et al., 1986). Therefore, it is possible that overexpressed L20 may accelerate the overall rate of ribosome assembly in the ESC19 strain, or that considering the fact that the binding site of BipA on 50S ribosomal subunit is remote from L20 on ribosome, L20 may partially stabilize the aberrant 50S particles (approximately 43S) in the ESC19 strain to complete 50S ribosomal subunit assembly.

Alternatively, it is also possible that, like BipA, the expression of *rplT* increases to promote and adjust 50S ribosomal subunit assembly under cold-shock stress conditions. The expression of *infC* encoding the translational initiation factor 3 (IF3) is enhanced from the strong cold-shock-responsive promoter (Giuliodori et al., 2007), which governs transcription of *rpmI* to *rplT*. Vigorous translation of *infC* at low temperature interferes with the formation of pseudoknot, which extends from the very 3′-end of *infC* to six bases upstream of the translation start site of *rpmI* (Chiaruttini et al., 1997). Consequently, L20-mediated *in trans* translational repression is derepressed, leading to increased L20 expression (Chiaruttini et al., 1997; Kocharunchitt et al., 2012). Therefore, it is likely that the expression of both *bipA* and *rplT* is modulated by cold-shock to support 50S ribosomal subunit biogenesis. Lastly, we do not rule out the possibility that other regulatory activity of L20 apart from ribosome assembly is involved in suppressing the deletion of *bipA*. L20 was found to be a potent enzyme inhibitor of ornithine decarboxylase *in vitro* (Kashiwagi and Igarashi, 1987), and it was one of two ribosomal proteins that disappear entirely from ribosomes but stay undiminished elsewhere in the cell during the postexponential growth of *E. coli* in L-broth (Ramagopal, 1984). This implies that L20 may be available for the interaction with other protein(s), which leads to the suppression under given conditions.

## Data Availabilty Statement

The raw data supporting the conclusions of this article will be made available by the authors, without undue reservation, to any qualified researcher.

## Author Contributions

EC designed the study, executed the experiments, analyzed the data, and wrote the manuscript. HJ assisted the experiments. J-IO reviewed the results and manuscript. JH supervised EC and HJ. All authors discussed the results and approved the final manuscript.

## Conflict of Interest

The authors declare that the research was conducted in the absence of any commercial or financial relationships that could be construed as a potential conflict of interest.
