# Supplementary material for: Overexpressed L20 Rescues 50S Ribosomal Subunit Assembly Defects of bipA-Deletion in Escherichia coli
Source: Front Microbiol. 2020 Jan 9;10:2982. doi: 10.3389/fmicb.2019.02982 (PMC6962249; doi:10.3389/fmicb.2019.02982)
Supplement: Supplementary file 1 [file Table_1.pdf]

Table S1. Probes used in northern blot analysis and hybridization and washing conditions

Probes	Sequence (5´→3´)	Hybridization (°C)	Washing (°C)	Reference
23S-M	AAGGTTAAGCCTCACGGTTC	60	57	(Charollais et al., 2003)
p23S-U	CGCTTAACCTCACAAC	50	47	(Charollais et al., 2003)
