# Supplementary material for: Overexpressed L20 Rescues 50S Ribosomal Subunit Assembly Defects of bipA-Deletion in Escherichia coli
Source: Front Microbiol. 2020 Jan 9;10:2982. doi: 10.3389/fmicb.2019.02982 (PMC6962249; doi:10.3389/fmicb.2019.02982)
Supplement: Supplementary file 2 [file Data_Sheet_1.PDF]

Figure S1

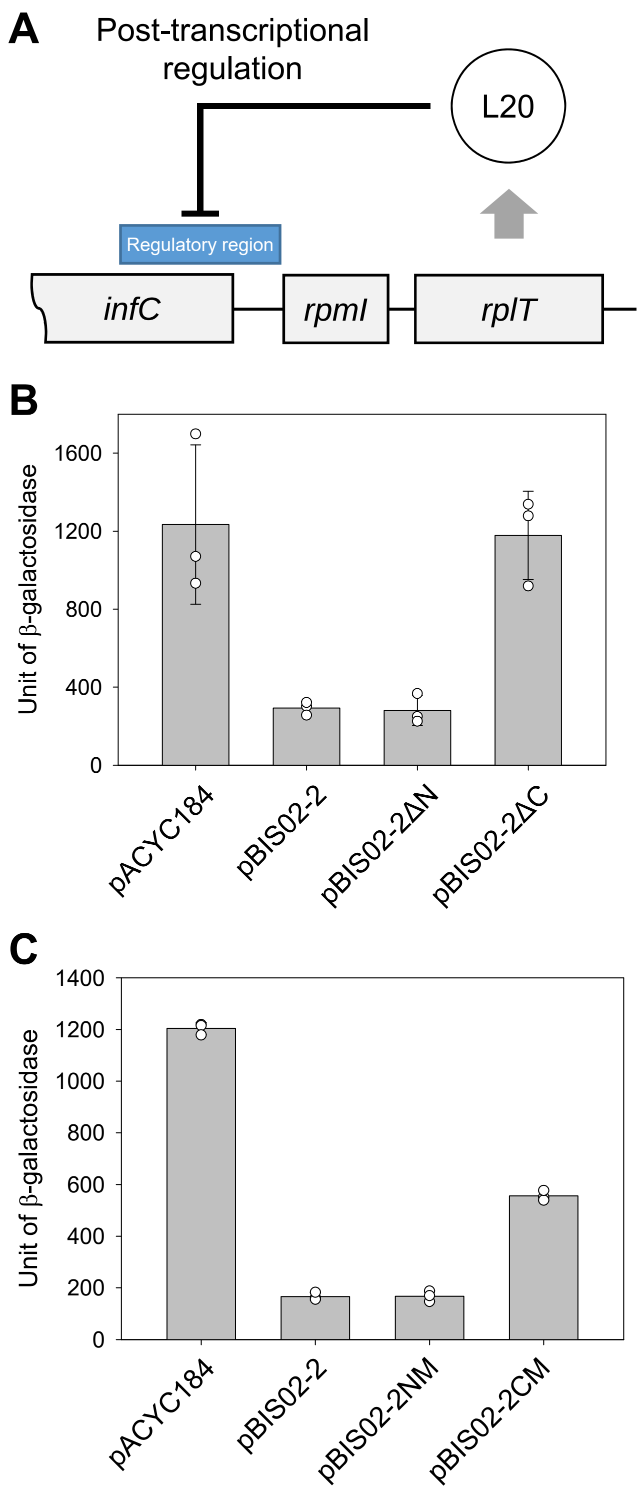

Figure S1. Regulatory activities of wild-type and mutated L20. **(A)** A model explaining L20-mediated repression of *rpmI* expression. Comparison of the effect of truncated mutations **(B)** and point mutations **(C)** on L20-mediated repression of *rpmI* expression. To measure the effect of mutations, ESC29 cells harboring pRS414-P_{rpmI} were transformed with pACYC184, pBIS02-2, pBIS02-2ΔN, pBIS02-2ΔC, pBIS02-2NM, or pBIS02-2CM and their β-galactosidase activities were measured as described in Materials and methods. Experiment was independently performed three times and each value is presented with the standard deviation.

Figure S2

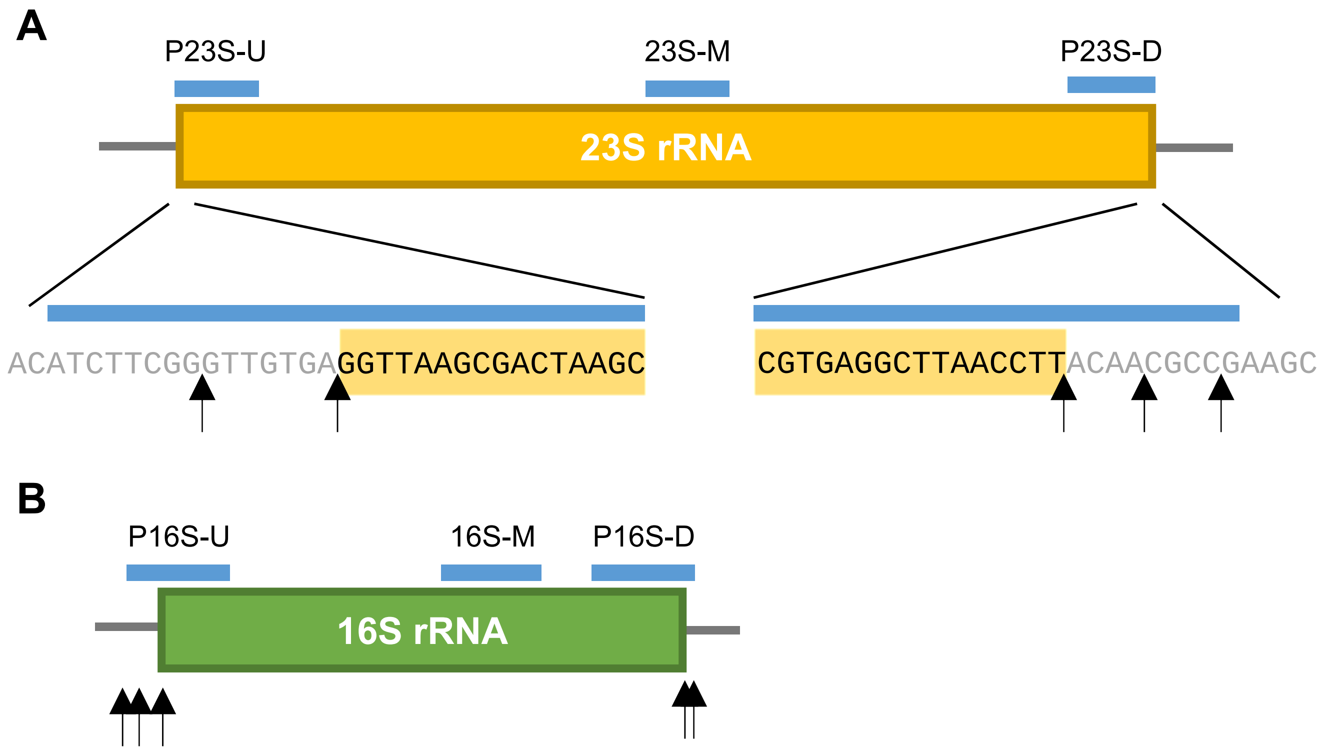

Figure S2. Schematics of the regions amplified by qRT-PCR on 23S **(A)** and 16S **(B)** rRNAs. The blue bars and arrows represent the amplified regions and processing sites, respectively.

Figure S3

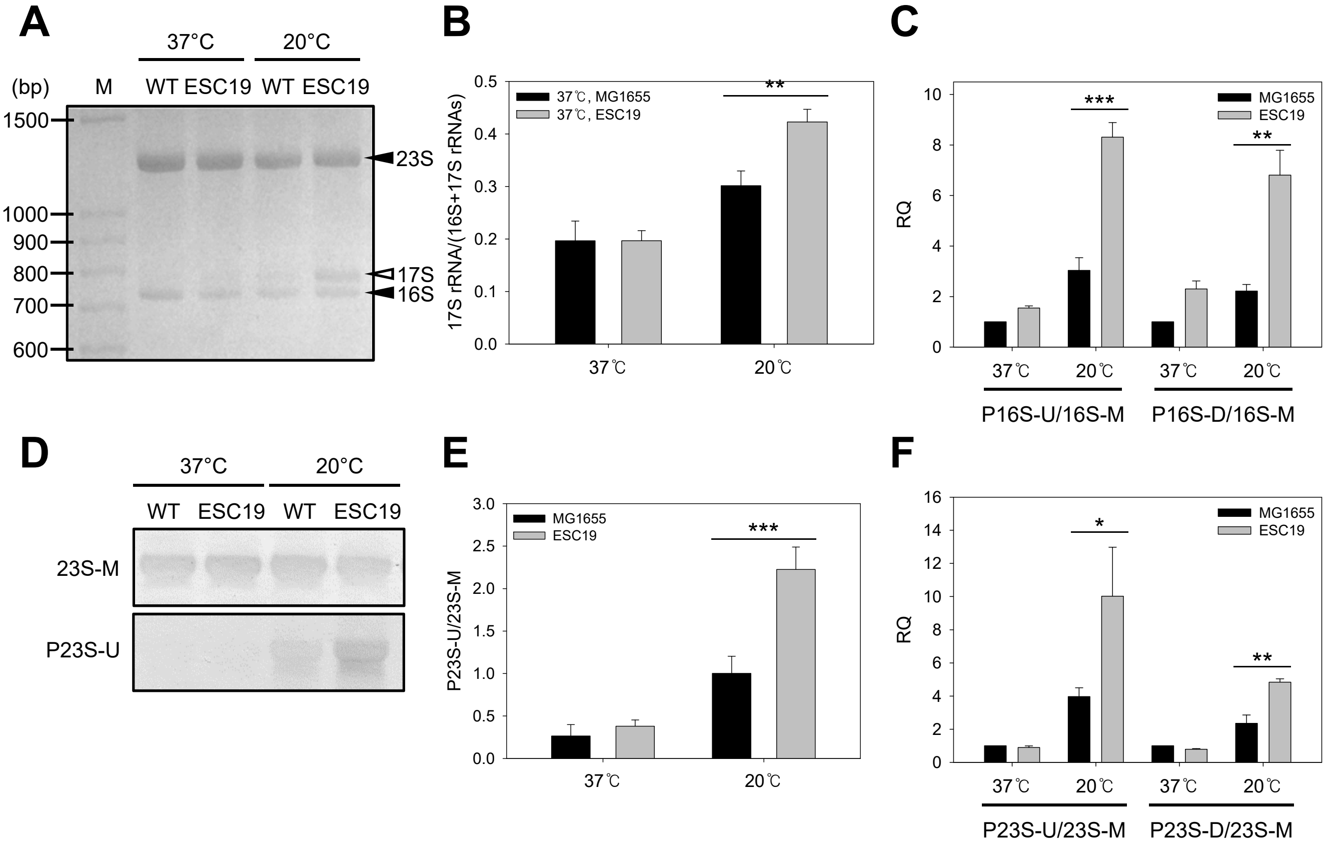

Figure S3. Effect of deletion of *bipA* on 23S and 16S rRNAs processing at low temperature. **(A)** Agarose gel electrophoresis of total RNAs in MG1655 (WT) and ESC19. 1 μg of total RNA was subjected to 2.0% agarose gel electrophoresis followed by ethidium bromide (EtBr) staining. Filled arrow heads indicate mature 16S and 23S rRNAs and open arrow head indicates 16S rRNA precursor (17S). M, 100-bp DNA ladder (TaKaRa). Experiments were performed in three independent repetitions. **(B)** Densitometric analysis of 16S rRNA processing. The intensity of band in Fig. S3A was measured using the band analysis tools of Image Lab software version 5.2.1 (Bio-Rad). Each column represents the mean value of three different experiments and error bars represent S.D. **(C)** qRT-PCR analysis of 16S rRNA processing in MG1655 and ESC19 grown at 37°C or 20°C. The unprocessed 16S rRNA values (P16S-U and -D) were normalized to the value of total 16S (16S-M). Experiments were performed in three independent repetitions as described in Materials and methods. **(D)** Northern blot analysis of 23S rRNAs extracted from wild-type MG1655 and ESC19. 4 μg of total RNA was separated on 1.2% agarose gel followed by transfer to a nylon membrane. rRNAs were detected by biotin-labelled probes described in Table S1. Northern blotting was performed in three independent repetitions. **(E)** Densitometric analysis of 23S rRNA processing. The gel analyzer function of Image J program was used for quantification of the blots in Fig. S3D. All data are presented as mean of three deferent experiments and error bars represent S.D. **(F)** qRT-PCR analysis of 23S rRNA processing in MG1655 and ESC19 grown at 37°C or 20°C. The immature 23S RNA values (P23S-U and -D) were normalized to the value of total 23S (23S-M). qRT-PCR was performed in three independent repetitions. Error bars represent S.D. Statistical significance was derived from the unpaired two-tailed t test. NS, nonsignificant; *, $p < 0.05$; **, $p < 0.01$; ***, $p < 0.001$.
